# Emerging Diversity of Channelrhodopsins and Their Structure-Function Relationships

**DOI:** 10.3389/fncel.2021.800313

**Published:** 2022-01-24

**Authors:** Elena G. Govorunova, Oleg A. Sineshchekov, John L. Spudich

**Affiliations:** Center for Membrane Biology, Department of Biochemistry and Molecular Biology, University of Texas Health Science Center at Houston McGovern Medical School, Houston, TX, United States

**Keywords:** microbial rhodopsins, ion channels, optogenetics, algae, phototaxis

## Abstract

Cation and anion channelrhodopsins (CCRs and ACRs, respectively) from phototactic algae have become widely used as genetically encoded molecular tools to control cell membrane potential with light. Recent advances in polynucleotide sequencing, especially in environmental samples, have led to identification of hundreds of channelrhodopsin homologs in many phylogenetic lineages, including non-photosynthetic protists. Only a few CCRs and ACRs have been characterized in detail, but there are indications that ion channel function has evolved within the rhodopsin superfamily by convergent routes. The diversity of channelrhodopsins provides an exceptional platform for the study of structure-function evolution in membrane proteins. Here we review the current state of channelrhodopsin research and outline perspectives for its further development.

## Introduction

Channelrhodopsins (ChRs) are microbial proteins that act as photoreceptors in photomotility of flagellate algae (Sineshchekov et al., 2002). ChRs are members of the rhodopsin superfamily, which comprises integral membrane proteins sharing common topology of seven transmembrane α-helices (TM1-TM7) and the retinal chromophore covalently bound *via* a Schiff base linkage to the ε-amino group of a specific lysine residue in TM7 (Spudich et al., 2000; Ernst et al., 2014; Kandori, 2020). According to their primary sequence homology and chromophore geometry, ChRs are type 1 rhodopsins (Govorunova et al., 2017a; Rozenberg et al., 2021), distinct from visual pigments (type 2 rhodopsins). So far type 1 rhodopsins are found only in microorganisms and their function is either photoenergy capture by light-driven ion pumping (Brown, 2014; Kandori, 2015) or photosensory signaling by three distinctly different mechanisms (Govorunova et al., 2017a): (i) participation as the photoactive subunit in sensory rhodopsin-transducer (SR-Htr) complexes in haloarchaea (Sasaki and Spudich, 2008); (ii) function as light-sensitive enzymes with guanylyl cyclase activity discovered in fungi (Avelar et al., 2014); and (iii) photocontrol of membrane electrical potential in algae (Sineshchekov et al., 2002). Each of these signaling processes controls flagellar motility, thereby mediating photomotility behavior in prokaryotes (i) or eukaryotic microorganisms (ii and iii).

Photoexcitation of ChRs results in passive ion transport across the membrane (Nagel et al., 2002, 2003). This unique light-gated channel activity, not found in any other natural proteins, enables stimulation or inhibition of specific cellular populations or even individual excitable cells with light on the submillisecond time scale, applying the transformative technique known as “optogenetics” (Boyden et al., 2005; Deisseroth, 2011). Recently, partial recovery of visual function in a human patient upon intraocular injection of an adeno-associated viral vector encoding a ChR was reported (Sahel et al., 2021). Further development of ChR-based gene therapy will depend on the availability of molecules with desired biophysical properties. Many excellent reviews discuss perspectives of using ChRs in biomedical research and therapy (Deisseroth, 2011, 2015; Lin, 2012; Yawo et al., 2013; Wiegert et al., 2017; Schneider-Warme, 2018). Here we focus on the molecules themselves and report recent advances on our understanding of their evolution, and structure-function relationships that determine biophysical characteristics of ChRs relevant for optogenetics: ionic selectivity, rectification, desensitization and color tuning.

## Convergent Evolution of Channelrhodopsins

An important insight gained from recent studies on ChR diversity is that channel function cannot always be deduced from the primary sequence alone, i.e., several ChR families exist that show very low sequence homology to each other yet similar function (Govorunova et al., 2021). A striking example is the family of cryptophyte CCRs known as “bacteriorhodopsin-like CCRs” (BCCRs; Sineshchekov et al., 2017). These proteins show higher sequence homology to haloarchaeal proton pumps than to chlorophyte CCRs, and yet passively conduct protons and metal cations (Govorunova et al., 2016b; Yamauchi et al., 2017; Marshel et al., 2019; Sineshchekov et al., 2020). In particular, their sequences retain the two carboxylate residues that define the vectorial proton path in bacteriorhodopsin, in which Asp85 and Asp96 serve as acceptor and donor, respectively, of the photoactive site Schiff base proton (Lanyi, 2006). In contrast, nearly all chlorophyte CCRs contain Glu in the first position, and a non-carboxylate residue (most frequently, His) in the second position. Analysis of laser flash-induced photocurrents and transient absorption changes in CCR2 from the cryptophyte *Guillardia theta* (*Gt*CCR2), a representative of BCCRs, has revealed a tight coupling between channel gating and intramolecular proton transfers involving the same residues that define vectorial proton transport in bacteriorhodopsin (Sineshchekov et al., 2017).

A high-resolution structure of a BCCR known as ChRmine, recently obtained by cryo-electron microscopy, revealed a trimeric organization (Kishi et al., 2021) typical of bacteriorhodopsin (Luecke et al., 1999). In contrast, X-ray structures of all other so far crystallized ChRs are homodimers (Kato et al., 2012; Volkov et al., 2017; Kim et al., 2018; Oda et al., 2018; Li et al., 2019; Zabelskii et al., 2020), confirmed by biophysical and biochemical analyses. Unlike canonical oligomeric channels, in which the channel pore is formed by several subunits, each ChR protomer is presumed to be conductive, and there is no evidence that dimerization plays any functional role. However, based on a combination of structural, functional and computational studies it has been suggested that ChRmine (and possibly other BCCRs) possess a cation conduction pathway along the trimer interface, in addition to that within individual monomers, as in other ChRs (Kishi et al., 2021).

## Taxonomic Distribution

**Figure 1:**
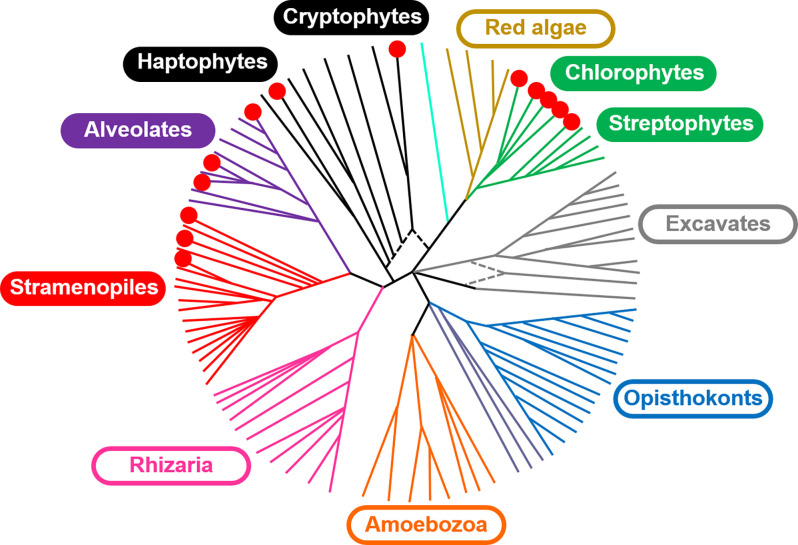
A schematic of the major lineages in the eukaryotic tree of life adapted from Keeling et al. (2014) distributed under the terms of the Creative Commons Public Domain declaration. The red circles indicate the lineages in which ChR homologs have been identified.

Exploration of natural ChR diversity may yield variants better suited for optogenetic applications than currently known molecules. But where should we look for new ChR sequences? The best-characterized *Chlamydomonas reinhardtii* ChRs serve as photoreceptors in phototaxis (Sineshchekov et al., 2002), so phototactic flagellates seem to be a promising hunting ground. Indeed, high-throughput polynucleotide sequencing projects have yielded >150 ChR homologs in the species of the class Chlorophyceae (Klapoetke et al., 2014; Rozenberg et al., 2020), to which *C. reinhardtii* belongs (Figure 1). ChR sequences were also found in the other three classes of the “core” chlorophytes, i.e., Chlorodendrophyceae, Trebouxiophyceae and Ulvophyceae, in *Pedinomonas minor* (class Pedinophyceae), and in the three classes of the polyphyletic group known as “prasinophytes” (Mamiellophyceae, Nephroselmidophyceae and Pyramimonadophyceae; Klapoetke et al., 2014; Rozenberg et al., 2020). ChR homologs have also been found in the several representatives of the phylum Streptophyta: the flagellate alga *Mesostigma viride* (Govorunova et al., 2011), included in Streptophyta as an early diverging lineage, the filamentous alga *Klebsormidium nitens* (Klebsormidiophyceae; Awasthi et al., 2020; Tashiro et al., 2021) and thallus-forming alga *Coleochaete irregularis* (Coleochaetophyceae; Rozenberg et al., 2020), the latter of which is considered as the closest relative of land plants (Leliaert et al., 2012). However, the sequenced genomes of land plants do not seem to encode ChRs (nor rhodopsins of any kind). Rhodopsin fragments reported in *Oryza sativa* most likely originate from fungal contamination (see discussion in Ruiz-Gonzalez and Marin, 2004).

Cryptophytes and dinoflagellates are two other algal lineages, representatives of which are capable of genuine phototactic orientation (Forward, 1974; Watanabe and Furuya, 1982; Erata et al., 1995; Horiguchi et al., 1999). More than 40 type I rhodopsin genes are predicted by the fully sequenced genome of the marine cryptophyte *G. theta* (Govorunova et al., 2015), at least 29 of which are expressed in native cells (Konno et al., 2020). Remarkably, in this alga and many other cryptophyte species analyzed by transcriptome sequencing projects there are two structurally and functionally distinct ChR families: ACRs and BCCRs (Klapoetke et al., 2014; Govorunova et al., 2015, 2016a,b, 2017b, 2018; Wietek et al., 2016; Yamauchi et al., 2017; Sineshchekov et al., 2020). Besides ChRs, cryptophyte genomes also encode type 1 rhodopsins of other families, such as the proton pump *Gt*R3 (Gradinaru et al., 2010). The absorption spectrum of *Gt*R1, the first rhodopsin identified in *G. theta* and purified upon expression of its gene in *E. coli*, overlapped with the action spectrum of *G. theta* photobehavior, so this protein was suggested as a candidate photoreceptor for this response (Sineshchekov et al., 2005). However, neither *Gt*R1 nor homologous *Gt*R2 generate channel currents in heterologous systems, so *G. theta* phototaxis is most likely guided by BCCRs and ACRs subsequently found in this organism.

As those of cryptophytes, the genomes of dinoflagellates encode multiple rhodopsins of different families. In particular, many rhodopsin transcripts were identified in the predatory dinoflagellate *Oxyrrhis marina* (Zhang et al., 2007; Slamovits et al., 2011). However, most *Oxyrrhis* rhodopsins are proteorhodopsin homologs (Rhiel et al., 2020). H^+^ pumping in one of them (OR1) was experimentally verified by heterologous expression followed by patch clamp recording (Janke et al., 2013). A group of rhodopsin sequences from dinoflagellates of the genera *Ansanella, Pelagodinum, Breviolum*, and *Symbiodinium* contain the Thr-Cys-Pro motif in the middle of TM3 that is conserved in most so far known ChRs. Two of such rhodopsins from the coral endosymbiont *Symbiodinium microadriaticum* exhibited channel activity upon heterologous expression (Govorunova et al., 2021). Their spectral sensitivity roughly matched that of phototactic accumulation observed in the Symbiodiniaceae and unclassified coral symbiotic dinoflagellates (Hollingsworth et al., 2005; Aihara et al., 2019), which suggests their photoreceptor role for this response. ChR-like sequences are also encoded by the genomes of the flagellate algae *Chromera velia* and *Vitrella brassicaformis* (Woo et al., 2015), representatives of the group Chromerida that belongs to the same clade Alveolata as dinoflagellates.

Functional ChRs have also been found in the haptophyte algae *Phaeocystis antarctica* and *P. globosa* (clade Prymnesiophyceae; Govorunova et al., 2020), although a non-homologous rhodopsin sequence from the haptophyte *Pavlova (Diacronema) lutheri* (order Pavlovaceae) was non-electrogenic upon heterologous expression (Klapoetke et al., 2014). Transcriptome sequencing within the frame of the 1,000 Plants (1KP) project (Matasci et al., 2014) has not identified rhodopsins in *Euglena gracilis.*

An important paradigm-shifting discovery of recent years has been that of ChRs in non-photosynthetic protists from the clade Stramenopiles. Labyrinthulea (also known as Labyrinthulomycetes) are a group of aquatic heterotrophic organisms that have independently evolved a fungus-like lifestyle (Pan et al., 2017). *Cafeteria roenbergensis* is a globally distributed marine bacterivorous flagellate that belongs to the same stramenopile class Bygira as Labyrinthulea (Hackl et al., 2020). ChRs from several Labyrinthulea species of the order Thraustochytrida, *C. roenbergensis* and the unclassified stramenopile strain TOSAG 23-3 have been functionally characterized by heterologous expression and patch clamp electrophysiology (Govorunova et al., 2020, 2021). ChRs from the latter source are highly homologous to metagenomically identified ChRs known as MerMAIDs (Metagenomically discovered, Marine, Anion-conducting and Intensely Desensitizing channelrhodopsins; Oppermann et al., 2019), which suggests that MerMAIDs also originate from stramenopiles. Most thraustochytrids produce flagellate zoospores, so the function of their ChRs in native cells is likely to be phototaxis, as that of algal ChRs. Indeed, phototaxis towards the light emitted by bioluminescent marine bacterium *Vibrio fischeri* has been documented in flagellate zoospores of the labyrinthulomycete *Ulkeni*a sp. that prey on these bacteria (Amon and French, 2004). The labyrinthulomycete* Aplanochytrium stocchinoi* does not produce flagellate zoospores (Moro et al., 2003), and yet its genome encodes ChR homologs (Rozenberg et al., 2020), the function of which is unknown. Very recently, functional ChRs have been identified in the *Hyphochytrium catenoides* from the stramenopile class Hyphochytriomycetes (e.g., Govorunova, O. A. Sineshchekov and J. L. Spudich, unpublished observations).

The high-throughput sequencing projects are a rich source of new ChR homologs, but the attribution of so identified sequences to particular organisms has to be treated with caution, even when axenic biomaterial is used. For example, a ChR sequence named ChRmine (Marshel et al., 2019) was found in the transcriptome of the ciliate *Tiarina fusus* within the frame of the Marine Microbial Eukaryote Transcriptome Sequencing Project (MMETSP; Keeling et al., 2014). However, this sequence is identical at the nucleotide level with a BCCR sequence derived from the cryptophyte *Rhodomonas lens* (Sineshchekov et al., 2020), which was used to feed *Tiarina* prior to RNA extraction. Similarly, other ChR homologs, attributed by MMETSP to the ciliates *Myrionecta rubra* and *Strombidinopsis* sp. (Rozenberg et al., 2020) are identical or very closely homologous to cryptophyte ChRs and almost certainly belong to the food organisms. The presence of rhodopsins in ciliates, based on retinal extraction and hydroxylamine inhibition experiments, has been suggested (Tokioka et al., 1991), but, to the best of our knowledge, no genetic evidence for this has so far been obtained.

ChR sequences are conspicuously absent in prokaryotes, most of which rely on enzymatic cascades for photosensory transduction (Armitage, 1997; Spudich, 2006). However, some eukaryotic ChR sequences are highly divergent, so it is plausible that prokaryotic ChRs simply could not be recognized by bioinformatic means alone. Patchy distribution of ChRs among eukaryotic taxa (Figure 1) suggests their acquisition by horizontal gene transfer and/or endosymbiosis. ChR genes found in giant viruses that infect marine protists (Rozenberg et al., 2020; Zabelskii et al., 2020) may represent one method of ChR transfer between different taxa, and endosymbiosis may represent another method. The origin of cryptophytes, dinoflagellates and haptophytes by secondary endosymbiosis (Hackett et al., 2004; Gentil et al., 2017) may explain a particularly large number of genes of different rhodopsin families in their genomes, transferred from the symbionts to the host nucleus.

## Permeation Pathway and Ion Selectivity

**Figure 2:**
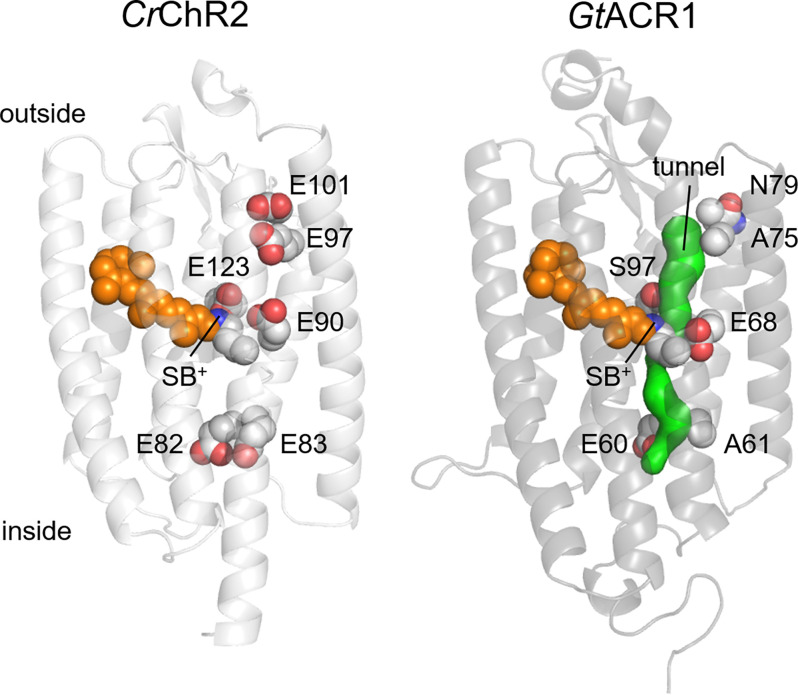
Crystal structures of *Cr*ChR2 (6EID; Volkov et al., 2017) and *Gt*ACR1 (6EDQ; Li et al., 2019). The side chains of six glutamates conserved in most CCRs and the chromophore are shown as spheres. The intramolecular tunnel is shown in green. SB^+^, protonated Schiff base.

In contrast to oligomeric ion channels, the selectivity of which is determined by a defined structural feature (called the “selectivity filter”), ion selectivity in ChRs appears to be controlled by several residues that form the ion conduction pathway within each protomer. No high-resolution structures of the open state are available yet for any ChR, but those of the closed state together with the results of functional studies strongly suggest that the channel pore is formed by TM1-3 and 7. In particular, *Gt*ACR1 structures (Kim et al., 2018; Li et al., 2019) reveal a narrow intramolecular tunnel connecting the cytoplasmic and extracellular aqueous phases (Li et al., 2019) that is expected to expand upon illumination (Figure 2). A bromide ion resolved near the cytoplasmic entry into this tunnel in the Br-soaked *Gt*ACR1 structure strongly confirms this hypothesis (Li et al., 2021). Crystal structures of chlorophyte CCRs reveal a series of unconnected intramolecular cavities in the dark state (Kato et al., 2012; Volkov et al., 2017; Oda et al., 2018). A time-resolved serial femtosecond crystallographic study using an X-ray free electron laser has shown an outward shift of TM3 and a local deformation of TM7 upon photoactivation of a hybrid chrolophyte CCR known as C1C2 (Oda et al., 2021).

Most chlorophyte CCRs contain five conserved Glu residues in TM2 and the TM2-TM3 loop along the predicted channel pore (Glu82, Glu83, Glu90, Glu97, and Glu101 in *Cr*ChR2; Figure 2). Mutagenetic studies have shown that Glu90 is required for cation conductance in *Cr*ChR2, as its replacement with Lys or Arg made the channel permeable for anions (Wietek et al., 2014), and its residual permeability for protons was eliminated by mutagenetic neutralization of two other glutamates (the E83Q and E101S mutations; Wietek et al., 2015). Alternatively, permeability for anions could be engineered in chlorophyte CCRs by multiple mutations in the channel pore reducing its overall negative charge (Berndt et al., 2014, 2016; Wietek et al., 2017). These results led to the notion that cation or anion selectivity of ChRs is determined by the electrostatic potential of the ion conducting pathway (Rappleye and Berndt, 2019).

**Figure 3:**
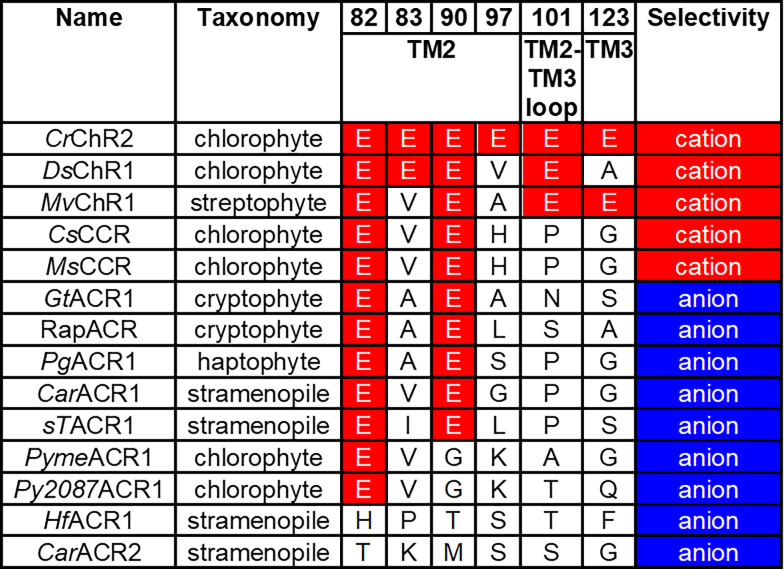
Residue motifs and ionic selectivity of representative ChRs. The residue numbers are according to the *Cr*ChR2 sequence. *Cr*ChR2, *Chlamydomonas reinhardtii* channelrhodopsin 2; *Ds*ChR1, *Dunaliella salina* channelrhodopsin 1; *Mv*ChR1, *Mesostigma viride* channelrhodopsin 1; *Cs*CCR, *Crustomastix stigmatica* cation channelrhodopsin; *Ms*CCR, *Mantoniella squamata* cation channelrhodopsin; *Gt*ACR1, *Guillardia theta* anion channelrhodopsin 1; RapACR, rapid anion channelrhodopsin from *Rhodomonas salina*; *Pg*ACR1, *Phaeocystis globosa* anion channelrhodopsin 1; *Car*ACR1, *Cafeteria roenbergensis* anion channelrhodopsin 1; *sT*ACR1, Stramenopiles sp. TOSAG23–3 anion channelrhodopsin 1; *Pyme*ACR1, *Pyramimonas melkonianii* anion channelrhodopsin 1; *Py*2087ACR1, *Pyramimonas* sp. CCMP2087 anion channelrhodopsin 1; *Hf*ACR1, *Hondaea fermentalgiana* anion channelrhodopsin 1; *Car*ACR2, *Cafeteria roenbergensis* anion channelrhodopsin 2.

Comparative analysis of wild-type CCRs and ACRs from different taxa supports this notion. In general, ACRs sequences contain a smaller number of conserved glutamates than do chlorophyte and streptophyte CCRs (Figure 3). However, the same residue pattern (Glu82 and Glu90 are conserved, and the other positions are occupied with non-carboxylate residues) is found in some chlorophyte CCRs and most ACRs from cryptophytes, haptophytes and stramenopiles. This observation suggests that the role of the glutamate residues in ion selectivity depends on a wider protein context.

Another residue that appears to be involved in discrimination between ACRs and CCRs is that in the position of Asp85, the primary acceptor of the Schiff base proton in bacteriorhodopsin. In all so far known ACRs this Asp is replaced with a non-carboxylate residue, whereas in nearly all chlorophyte CCRs this carboxylate is conserved as Glu (and as Asp in BCCRs). However, this residue alone cannot be regarded as the sole indicator of anion selectivity, because some chlorophyte CCRs also contain a non-carboxylate residue in this position [e.g., *Ds*ChR1 from *Dunaliella salina* (Zhang et al., 2011) and Chronos from *Stigeoclonium helveticum* (Klapoetke et al., 2014)]. Mutagenetic neutralization of this residue (Glu123) in *Cr*ChR2 accelerated channel opening, but did not convert the cation channel into an anion channel (Gunaydin et al., 2010).

CCRs of both chlorophyte and cryptophyte families differ in their Na^+^/H^+^ permeability ratios. Among chlorophyte CCRs, ChR1 from *M. viride* (*Mv*ChR1) and ChR2 from *Platymonas (Tetraselmis) subcordiformis* (*Ps*ChR2) exhibit a higher relative permeability to Na^+^ than *Cr*ChR2 (although they still are primarily H^+^ channels; Govorunova et al., 2011, 2013; Duan et al., 2019). Most so far tested cryptophyte BCCRs also have an increased Na^+^/H^+^ permeability ratio (e.g., ~5 × 10^−5^ in *Gt*CCR4), as compared to *Cr*ChR2 (~10^−6^) (Govorunova et al., 2016b; Shigemura et al., 2019). A number of chlorophyte and cryptophyte CCRs are almost purely H^+^ channels (Berthold et al., 2008; Tsunoda and Hegemann, 2009; Zhang et al., 2011; Govorunova et al., 2021). Some CCRs are also permeable for Ca^2+^, although to a lesser extent than for monovalent metal cations, e.g., the Ca^2+^/Na^+^ permeability ratio of *Cr*ChR2 is ~0.15 (Kleinlogel et al., 2011).

## Rectification

For a simple aqueous pore in a lipid bilayer, the relationship between the membrane voltage and transmembrane current (the current-voltage relationship, or the IE curve) is linear as defined by Ohm’s law. However, many ion channels show deviation from this linearity known as “rectification” (Hille, 2001). A likely reason for such behavior would be the difference in the concentrations of the permeated ions on the two sides of the membrane, as modeled by the Goldman-Hodgkin-Katz current equation (Hille, 2001), but many ion channels, including ChRs, show rectification even when probed under symmetrical ionic conditions.

Peak photocurrent generated by *Gt*ACR1 showed a linear dependence on voltage (Govorunova et al., 2015). A protein-wide Glu substitution screen has revealed that introducing a negative charge in the cytoplasmic half of the presumed ion pathway caused outward rectification, whereas that in the extracellular half, inward rectification (Sineshchekov et al., 2019). Wild-type *Gt*ACR1 photocurrents decay biphasically even under single-turnover conditions (Sineshchekov et al., 2015). The amplitude of the fast decay component shows outward, and that of the slow decay component, inward rectification. Ala61 (corresponding to Glu83 of *Cr*ChR2) is located on the cytoplasmic surface near the entry into the channel pore, and Ala75 (corresponding to Glu97 of *Cr*ChR2), near the extracellular entry into the channel pore (Figure 2). The A61E mutation enhanced the rate and contribution of the fast channel closing, whereas the A75E mutation enhanced the slow component. A similar correlation between rectification and photocurrent kinetics was observed in the outwardly rectifying Q46E and G242E mutants and the inwardly rectifying G80E and S227E mutants. In wild-type *Gt*ACR1 the fast channel closing component temporally correlated with the formation of the M intermediate of the photochemical cycle (i.e., deprotonation of the Schiff base), and the slow closing component, with M decay (Sineshchekov et al., 2016). This correlation also held for the A61E and A75E mutants, which suggests that an acidic group placed near the cytoplasmic or extracellular entry into the channel electrostatically influences the photoactive site.

The IE curves of most chlorophyte CCRs and cryptophyte BCCRs show inward rectification (Nagel et al., 2002, 2003; Zhang et al., 2008; Govorunova et al., 2011, 2016b; Hou et al., 2012; Yamauchi et al., 2017), which is not caused by voltage-dependent block by cytoplasmic Mg^2+^ (Gradmann et al., 2011), as in some oligomeric K^+^ channels (Nichols et al., 1994). Quantitative modeling of the IE curves for *Cr*ChR2 using the formalism of enzyme kinetics led to the conclusion that inward rectification results from a combination of a nonlinear transport function and asymmetric competition between several cation species (Gradmann et al., 2011). The addition of Gd^3+^ or La^3+^ to the bath decreased inward rectification in CCRs, which was explained by a more selective block of cationic influx rather than efflux (Tanimoto et al., 2013). The residue in the Glu97 position of *Cr*ChR2 near the extracellular end of TM2 contributed to the Gd^3+^ effect (Tanimoto et al., 2013; Watanabe et al., 2016).

## Desensitization

When ChRs are activated with pulses of continuous light, their photocurrents first rise and then decline to a lower steady-state level even if the light is still on (the phenomenon known as desensitization). Desensitization reflects formation of an equilibrium mixture of photocycle intermediates and in different ChRs varies from nearly zero to practically 100% in MerMAIDs (Oppermann et al., 2019) and some *Rhodomonas* BCCRs (Sineshchekov et al., 2020). In a particular ChR, desensitization depends on the wavelength and intensity of the light (Ishizuka et al., 2006), membrane voltage (Ernst et al., 2008; Oppermann et al., 2019) and pH (Hegemann et al., 2005; Tsunoda and Hegemann, 2009), as all these factors are known to influence the photocycle.

Strong desensitization is a problem for most optogenetic applications, so elucidation of its molecular mechanisms is an important direction of ChR research. Theoretically, accumulation of any long-lived non- or less-conductive intermediate of the photocycle would result in desensitization. Experimental evidence shows that these intermediates are different in different ChR families. In *Cr*ChR2 desensitization is correlated with accumulation of the P480 state that is considered either as a late intermediate in a single branched photocycle (Lorenz-Fonfria and Heberle, 2014; Saita et al., 2018) or the initial state of a parallel photocycle that is formed in the primary photoreaction of the initial dark state (Kuhne et al., 2019). A recent study using DNP (dynamic nuclear polarization) enhanced solid-state MAS (magic-angle spinning) NMR spectroscopy has shown that P480 contains 13-*cis*, 15-*syn* retinal Schiff base and occurs late in the photocycle (Becker-Baldus et al., 2021). To return to the dark state, this chromophore configuration has to change, which could explain the long lifetime of the desensitized state. In MerMAID1 desensitization results from accumulation of the M intermediate (Oppermann et al., 2019), and in similarly rapidly desensitizing *Rhodomonas* BCCRs, from formation of an additional extremely blue-shifted intermediate P330 (Sineshchekov et al., 2020).

The E44Q and C84T mutations reduced desensitization in MerMAID1 (Oppermann et al., 2019). However, the mutated residues (corresponding, respectively, to Glu90 and Cys128 of *Cr*ChR2) are conserved in many ChRs that do not show strong desensitization, and therefore cannot be the sole cause of strong desensitization in MerMAIDs. As in the case of ion selectivity, the phenotypic effect of a particular residue substitution depends on a wider protein context.

## Color Tuning

Most optogenetic applications benefit from red-shifted ChRs, because light of longer wavelengths is less scattered by biological tissues resulting in a longer penetration depth. Combinatorial (multiplex) optogenetics, such as simultaneous activation (or inhibition) of two different neuronal populations in the same preparation, or combination of neuronal activation/inhibition with imaging of neuronal activity, requires both blue- and red-shifted ChRs with narrow absorption bands to avoid spectral overlap. Many individual residues (primarily in the retinal-binding pocket) have been shown to control the absorption wavelength in ChRs. However, their mutations intended to produce a spectral shift are usually associated with reduction of the photocurrent amplitude and/or deceleration of the photocurrent kinetics. Therefore, identification of natural spectrally shifted ChR variants, optimized by evolution, is particularly important.

**Figure 4:**
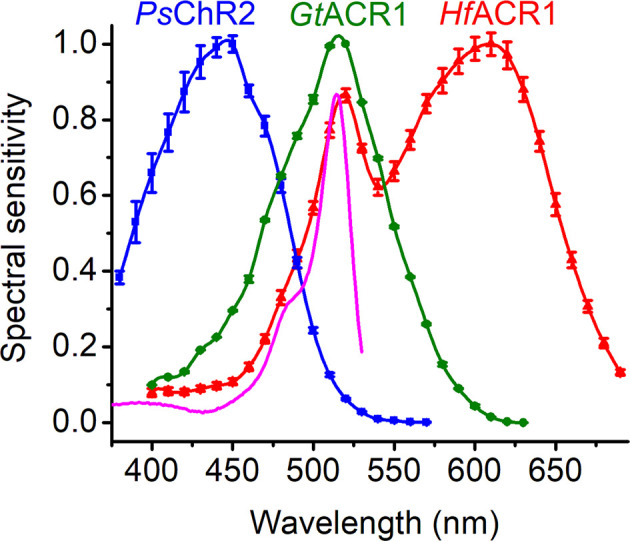
The action spectra of photocurrents generated by representative ChRs tagged with EYFP. The magenta line shows the absorption spectrum of EYFP. Note the additional band in the *Hf*ACR1 spectrum that reflects FRET from EYFP to rhodopsin. *Ps*ChR2, *Platymonas subcordiformis* channelrhodopsin 2; *Gt*ACR1, *Guillardia theta* anion channelrhodopsin 1; *Hf*ACR1, *Hondaea fermentalgiana* anion channelrhodopsin 1.

A strongly red-shifted CCR variant from *Chlamydomonas noctigama* named Chrimson has already been known for several years (Klapoetke et al., 2014; Oda et al., 2018), but its counterparts among ACRs have been identified only recently (Govorunova et al., 2020). Four ACRs, found in Labyrinthulea and collectively designated as RubyACRs, generate large photocurrents with the most red-shifted action spectra of any microbial rhodopsins known so far (up to 610 nm in *Hondaea fermentalgiana* anion channelrhodopsin 1 (*Hf*ACR1), the spectrum of which is shown in Figure 4, red). Their retinal-binding pockets show a distinctly different residue pattern as compared to that of Chrimson, indicating that the red spectral shift is achieved in RubyACRs by a different biophysical mechanism. Yet another residue pattern was found in two prasinophyte CCRs with the spectra red-shifted to 585 nm (Govorunova et al., 2021).

The isomer composition and stability of the chromophore also contribute to color tuning in ChRs. For example, according to the crystal structure, the polar S atom of the Cys102 sidechain in *Gt*ACR1 is oriented towards the Schiff base, which destabilizes the singlet excited state S_1_ (Tsujimura et al., 2021). Replacement of this Cys with a non-polar Ala is expected to red-shift the spectrum, but a 10-nm blue shift has been observed experimentally (Govorunova et al., 2018). Chromatographic analysis revealed an increased fraction of the blue-shifted 13-*cis* isomer in the C102A mutant as compared to the wild type, which suggests a decreased stability of the all-*trans* retinal configuration in the unphotolyzed state of this mutant (Tsujimura et al., 2021).

The ring/chain coplanarity of the chromophore is an important steric factor in color tuning. Mutations of the binding pocket residues that stabilized the twisted chromophore conformation led to the blue shift of the peak absorption from 476 to 455 nm in C1C2 (Kato et al., 2015). Two closely related CCRs, *Ps*ChR2 (Govorunova et al., 2013) and *Ts*ChR from *Tetraselmis striata* (Klapoetke et al., 2014), are the most blue-shifted ChRs known to date (Figure 4). In these proteins, the positions of Thr198 and Gly202 of C1C2 are occupied by Gly and Ala, respectively. Such a residue pattern strongly suggests that their chromophore is fixed in the twisted 6-*s*-*cis* configuration, which contributes to their blue-shifted absorption.

Microbial rhodopsins with absorption in the UV spectral region have been found among histidine kinase rhodopsins (HKRs), which do not generate transmembrane ionic currents, but exhibit an intrinsic enzymatic function (Mukherjee et al., 2019). The best studied representative, *C. reinhardtii* HKR1, binds 13-*cis*, 15-*anti* retinal *via* an unprotonated Schiff base (Luck et al., 2012; Penzkofer et al., 2014) and undergoes a unique dual isomerization upon photoexcitation (Hontani et al., 2020). At present it is unclear whether such photochemistry can support channel function.

Finally, the absorption spectra of ChRs can be shifted by replacement of the natural chromophore with natural or synthetic analogs. 3,4-dehydroretinal, also known as A2 retinal, naturally occurs in the eyes of some animals and has an additional double bond in the ring, which extends its π-conjugated system and red-shifts the absorption spectrum. Incorporation of all-*trans* A2 retinal shifted the action spectra of photocurrents generated by chlorophyte CCRs (Sineshchekov et al., 2012) and RubyACRs (Govorunova et al., 2020) 10–40 nm to longer wavelengths without significant effects on photocurrent kinetics. In contrast, a red-shifting effect of a synthetic analog with a modified ring (dimethylamino-retinal, or DMAR) was accompanied by deceleration of the channel kinetics in *Cr*ChR2 mutants (Azimi Hashemi et al., 2014). Extension of the chromophore π-system can alternatively be achieved by elongation of the polyene chain upon the insertion of a vinylene group. A series of such analogs has been tested in purified CCRs mutated to improve their affinity for such compounds (Shen et al., 2018; Okitsu et al., 2020). Channel activity of these molecules has not yet been tested, but measurements of flash-induced absorbance changes have revealed that the kinetics of the photocycle was altered, as compared to that of all-*trans* retinal-bound proteins.

The constructs used for heterologous expression of ChRs in animal cells usually encode fluorescent proteins as tags. Provided there is sufficient spectral overlap between the tag and ChR, Förster resonance energy transfer (FRET) occurs, which is manifested by an additional band or shoulder in the action spectrum of photocurrents. Such bands have been reported for C-terminal EYFP fusions of RubyACRs (Figure 4) and red-shifted chlorophyte CCRs (Govorunova et al., 2021), and can be expected in other cases as well. A contribution of FRET explains some discrepancies in the reported action spectra of photocurrents by the same ChR. For example, an EYFP (absorption peak at 513 nm) fusion of *Psu*ACR1 from *Proteomonas sulcata* exhibited peak sensitivity at ~520 nm (Govorunova et al., 2016a), as compared to that of a GFP (absorption peak at 485 nm) fusion with the peak at 540 nm (Wietek et al., 2016). The addition of fluorescent tags serving as FRET donors might be used as an engineering strategy to design tools for optogenetic applications in which a broad-band efficiency is desired, as has previously been achieved by fusing two ChRs with different spectra (Batabyal et al., 2015).

## Perspectives

Rapid progress in ChR research in recent years suggests that neither their natural diversity, nor molecular mechanisms have been fully explored. Better understanding of these mechanisms has not only fundamental, but also practical importance for improving their utility as optogenetic tools, e.g., obtaining more selective light-gated channels. In particular, a purely Na^+^-selective CCR would eliminate photoinduced acidification of the cytoplasm (Lin et al., 2009) that may activate various endogenous ion channels and receptors (Beppu et al., 2014), and an elevation of the cytosolic Ca^2+^ (Lin et al., 2009) may cause release of Ca^2+^ from intracellular stores and influence the activity of cellular kinases and transcription factors. On the other hand, a purely Ca^2+^-selective CCR would be invaluable for photocontrol of many Ca^2+^-regulated physiological and biochemical processes such as synaptic release, and a K^+^-selective CCR would be a powerful optogenetic tool for neuronal silencing.

So far, electrophysiological characterization has lagged behind bioinformatic identification of new ChR sequences. The advent of automated patch clamping is likely to change that (Suk et al., 2019; Obergrussberger et al., 2021). Recently, a high-throughput, automated planar patch clamp system has been used to determine ion permeability of novel cryptophyte ACR homologs (Govorunova et al., 2021). In this approach, a suspension of dissociated cells is loaded into wells of a 384-well plate with a glass bottom, in which microscopic orifices are bored. Suction is applied to capture a cell in each well and form a gigaohm seal. This technique allows simultaneous recording from 384 or even 768 cells (in two-module systems), which greatly reduces the time of experimentation. Alternatively, fluorescent voltage, proton and/or Ca^2+^ imaging can be used for rapid screening of multiple CCR variants and mutants (Cho et al., 2019).

Rational protein engineering can improve biophysical properties of ChRs to address specific needs of optogenetic applications. However, mutations that bring about desired changes such as faster current kinetics or red-shifted absorption are usually accompanied by undesired effects, such as reduction of expression and/or conductance. Machine learning algorithms might be a means to solve this problem. They have already been used successfully to generate chimeric ChRs (Bedbrook et al., 2017), and to improve expression, plasma membrane localization, and light sensitivity of ChRs (Bedbrook et al., 2019). Machine learning has also been employed to predict and tune spectral properties of ion-pumping microbial rhodopsins (Inoue et al., 2021), and the same procedures can be applied to ChRs. A systematic analysis of already identified ChR variants and mutants is needed to produce a reliable training set for machine learning.

## Author Contributions

EG, OS, and JS conceived the study and wrote the manuscript. All authors contributed to the article and approved the submitted version.

## Conflict of Interest

The authors declare that the research was conducted in the absence of any commercial or financial relationships that could be construed as a potential conflict of interest.

## Publisher’s Note

All claims expressed in this article are solely those of the authors and do not necessarily represent those of their affiliated organizations, or those of the publisher, the editors and the reviewers. Any product that may be evaluated in this article, or claim that may be made by its manufacturer, is not guaranteed or endorsed by the publisher.
